# Association between PUQE-based severity of hyperemesis gravidarum and systemic inflammatory indices

**DOI:** 10.1186/s12884-026-08949-x

**Published:** 2026-03-14

**Authors:** Aykut Kından, Belgin Savran Üçok

**Affiliations:** 1 Department of Obstetrics and Gynecology, Pursaklar State Hospital, Mimar Sinan, Çağatay Cd No:39, Ankara, Pursaklar 06145 Turkey; 2 Department of Obstetrics and Gynecology, Etlik City Hospital, Ankara, Turkey

**Keywords:** Hyperemesis gravidarum, PUQE score, Systemic inflammation, Systemic immune-inflammation index, Neutrophil-to-lymphocyte ratio, Hospitalization

## Abstract

**Objective:**

To evaluate the relationship between systemic inflammatory indices and the severity of hyperemesis gravidarum (HG) using the Pregnancy-Unique Quantification of Emesis (PUQE) score, and to assess their predictive value for hospitalization.

**Methods:**

This prospective case–control study included 80 first-trimester pregnant women: 40 with HG and 40 healthy controls. Demographic, hematological, and biochemical data were analyzed. Systemic inflammatory indices—neutrophil-to-lymphocyte ratio (NLR), monocyte-to-lymphocyte ratio (MLR), platelet-to-lymphocyte ratio (PLR), and systemic immune-inflammation index (SII)—were calculated from complete blood counts. Correlations between PUQE score, urinary ketone levels, and these indices were assessed using Spearman’s test, and ROC analysis was used to determine predictive performance for hospitalization.

**Results:**

Compared with controls, HG patients had significantly higher PUQE scores, white blood cell, neutrophil, monocyte, and platelet counts, and lower hemoglobin and sodium levels (all *p* < 0.05). NLR, MLR, PLR, and SII were markedly elevated in HG (all *p* < 0.001). PUQE and urinary ketone levels correlated positively with NLR (*r* = 0.703 and 0.786), MLR (*r* = 0.415 and 0.503), PLR (*r* = 0.469 and 0.563), and SII (*r* = 0.746 and 0.832) (all *p* < 0.001). SII showed the highest diagnostic accuracy for hospitalization (AUC = 0.973, 95% CI: 0.924–1.000).

**Conclusion:**

Systemic inflammatory indices, particularly SII and NLR, are significantly associated with the clinical severity of hyperemesis gravidarum and may serve as supportive markers for identifying patients at increased risk of hospitalization. These easily obtainable and inexpensive hematological markers may aid in identifying patients at higher risk who require inpatient management.

## Introduction

Hyperemesis gravidarum (HG) represents the most severe form of nausea and vomiting during pregnancy, affecting approximately 0.3–2% of all pregnancies [1, 2]. Unlike the self-limited and mild nature of common morning sickness, HG is characterized by persistent nausea, intractable vomiting, dehydration, ketonuria, and electrolyte imbalance, which may result in hospitalization and maternal–fetal complications [3, 4]. Although its pathophysiology remains incompletely understood, hormonal, genetic, and immunologic factors have been proposed to play a role in its development.

Recent research has increasingly suggested that systemic inflammation may contribute to the pathogenesis and severity of HG [5, 6]. Pregnancy itself induces a controlled inflammatory state; however, in HG, an excessive immune or inflammatory response might disturb this balance [7]. Various hematological markers, derived from complete blood count parameters—such as the neutrophil-to-lymphocyte ratio (NLR), platelet-to-lymphocyte ratio (PLR), monocyte-to-lymphocyte ratio (MLR), and the systemic immune-inflammation index (SII)—have emerged as inexpensive and practical indicators of systemic inflammation in different clinical contexts [8, 9]. These indices were selected because they are readily obtainable from routine laboratory testing, cost-effective, and reproducible, allowing potential integration into daily clinical decision-making without additional diagnostic burden.

Previous studies investigating the relationship between these indices and HG have reported inconsistent results, partly due to differences in disease severity classification and patient selection criteria [10]. The Pregnancy-Unique Quantification of Emesis (PUQE) scoring system provides an objective, standardized tool to assess the severity of nausea and vomiting, making it particularly suitable for correlating clinical severity with laboratory parameters [11]. However, data correlating PUQE-based severity grading with systemic inflammatory indices in HG remain limited. Moreover, few studies have comparatively evaluated multiple hematological inflammatory indices in relation to both standardized severity grading and hospitalization risk, a clinically meaningful outcome.

Therefore, the present study aimed to evaluate the relationship between PUQE score–based disease severity and systemic inflammatory indices (NLR, PLR, MLR, and SII) in patients with hyperemesis gravidarum, and to compare these parameters with healthy pregnant controls.

## Materials and methods

### Study design and setting

This prospective case–control study was conducted at the Department of Obstetrics and Gynecology, Pursaklar State Hospital (Ankara, Türkiye) between June 15, 2025 and October 1, 2025. The study was designed to investigate the relationship between the severity of hyperemesis gravidarum (HG), assessed by the Pregnancy-Unique Quantification of Emesis (PUQE) score, and systemic inflammatory indices derived from complete blood count (CBC) parameters. Ethical approval was obtained prior to the initiation of the study from the Institutional Ethics Committee of Pursaklar State Hospital (Decision No: 294, dated June 11, 2025). Participant recruitment and data collection commenced only after formal ethical approval was granted. All procedures were conducted in strict accordance with the ethical principles of the Declaration of Helsinki (2013 revision). Written informed consent was obtained from all participants before enrollment.

### Study population

A total of 80 pregnant women in the first trimester (6–14 weeks of gestation) were enrolled in this prospective case–control study, including 40 patients diagnosed with hyperemesis gravidarum (HG) and 40 healthy pregnant controls. The control group was recruited consecutively from women attending routine first-trimester antenatal visits at the same institution during the study period. All control participants underwent the same clinical evaluation and laboratory testing protocol as the HG group. The same exclusion criteria were applied to both groups to ensure comparability. Controls had no history of hyperemesis gravidarum in the current pregnancy and required no medical intervention for nausea or vomiting.

#### Diagnosis and eligibility criteria

The diagnosis of HG was based on persistent nausea and vomiting associated with dehydration, weight loss greater than 5% of pre-pregnancy weight, ketonuria, or electrolyte imbalance. The severity of HG was quantified using the Pregnancy-Unique Quantification of Emesis (PUQE) score, categorized as mild (≤ 6), moderate [7–12], or severe (≥ 13). Healthy pregnant women with no or minimal nausea and vomiting (PUQE < 3), matched for age, gestational week, and body mass index (BMI), were included as the control group.

#### Exclusion criteria

Participants with chronic systemic diseases (hypertension, diabetes mellitus, thyroid disorders, autoimmune or rheumatologic conditions), hepatic or renal impairment, multiple pregnancies, fetal anomalies, pregnancy complications such as preeclampsia or gestational diabetes, acute or chronic infections, or those receiving corticosteroids, anti-inflammatory, or immunosuppressive medications were excluded from the study.

### Data collection and laboratory analysis

Demographic and obstetric characteristics—including age, body-mass index (BMI), gravidity, parity, abortion history, and gestational age—were recorded at admission. Venous blood samples were collected after overnight fasting on the first morning of hospital evaluation, prior to any intravenous therapy.

CBC parameters (white blood cell count (WBC), neutrophil, lymphocyte, monocyte, and platelet counts, hemoglobin, and hematocrit) were measured using an automated hematology analyzer (Sysmex XN-1000, Kobe, Japan). Biochemical analyses—including sodium (Na), potassium (K), chloride (Cl), aspartate aminotransferase (AST), alanine aminotransferase (ALT), and lactate dehydrogenase (LDH)—were performed using standard enzymatic methods on a Beckman Coulter AU5800 analyzer. Urine ketone levels were semiquantitatively evaluated via dipstick test and categorized as *negative*,* +*,* ++*,* +++*. The following systemic inflammatory indices were calculated from CBC parameters: NLR = Neutrophil / Lymphocyte; MLR = Monocyte / Lymphocyte; PLR = Platelet / Lymphocyte; SII = (Platelet × Neutrophil) / Lymphocyte. All computations were automatically verified in the digital dataset to ensure accuracy and consistency.

### Hospitalization criteria

Patients were hospitalized when clinical signs of dehydration, electrolyte imbalance, or inability to tolerate oral intake were present despite outpatient management. Specifically, hospitalization was indicated in the presence of one or more of the following findings: persistent vomiting leading to weight loss greater than 5% of pre-pregnancy weight, ketonuria of + + or higher on urinalysis, signs of dehydration such as dry mucous membranes, tachycardia, or orthostatic hypotension, laboratory evidence of electrolyte disturbance (hyponatremia, hypokalemia, or metabolic alkalosis), and failure of oral or outpatient antiemetic therapy. During hospitalization, patients received intravenous fluid replacement, correction of electrolyte imbalances, and parenteral or oral antiemetic therapy as clinically indicated.

### Outcomes

The primary outcome of the study was the association between systemic inflammatory indices (NLR, MLR, PLR, and SII) and PUQE-based disease severity in hyperemesis gravidarum. The secondary outcome was the evaluation of these inflammatory indices in relation to hospitalization status within the HG group. Hospitalization criteria were predefined prior to data collection, and subgroup comparisons were conducted according to this prospectively defined clinical outcome.

### Statistical analysis

All statistical analyses were performed using IBM SPSS Statistics for Windows, Version 27.0 (IBM Corp., Armonk, NY, USA). The normality of data distribution was assessed using the Shapiro–Wilk test. Continuous variables were expressed as mean ± standard deviation (SD), and categorical variables as numbers and percentages (%). Comparisons between two independent groups (HG vs. control; hospitalized vs. non-hospitalized HG) were made using the independent samples *t*-test for normally distributed data and the Mann–Whitney *U* test for non-normally distributed data. Categorical variables were analyzed using the chi-square (χ²) test or Fisher’s exact test, as appropriate. Correlation analyses between PUQE score, urinary ketone levels, and systemic inflammatory indices (NLR, MLR, PLR, and SII) were performed using Spearman’s correlation test. To evaluate the diagnostic performance and determine optimal cut-off values of inflammatory markers for predicting hospitalization, receiver operating characteristic (ROC) curve analysis was conducted. The optimal cut-off values were determined using the Youden index (J = sensitivity + specificity − 1), selecting the threshold that maximized the combined sensitivity and specificity. A p-value < 0.05 was considered statistically significant for all analyses. A post hoc power analysis was conducted using G*Power (version 3.1, Heinrich Heine University Düsseldorf, Germany) to evaluate the statistical sensitivity of the primary between-group comparisons. Power was calculated for a two-tailed independent-samples t-test with α = 0.05. Effect size (Cohen’s d) was derived from the observed group means and standard deviations. For the HG versus control comparison, the observed effect size for SII was d = 1.77 with *n* = 40 per group (df = 78), yielding a noncentrality parameter (δ) of 7.92 and an achieved power of 0.94.

## Results

There were no significant differences between the hyperemesis gravidarum (HG) and control groups regarding age, body mass index (BMI), parity, abortion history, or gestational age (*p* > 0.05). However, the PUQE score was markedly higher in the HG group (10.2 ± 1.9) than in controls (2.7 ± 1.7; *p* < 0.001). Among hematological parameters, white blood cell (WBC), neutrophil, monocyte, and platelet counts were significantly elevated in HG patients (all *p* < 0.001), while hemoglobin and hematocrit values were lower compared with controls (*p* = 0.006 and *p* < 0.001, respectively). Serum sodium and potassium levels were significantly lower in the HG group (*p* < 0.001 and *p* = 0.028), whereas AST, ALT, and LDH were higher (all *p* < 0.05). Regarding inflammatory indices, NLR, MLR, PLR, and SII values were all significantly increased in HG patients compared with healthy pregnancies (all *p* < 0.001) (Table 1).

**Table 1:** Baseline Clinical and Laboratory Characteristics in HG and Control Groups

	Control	HG	
	Mean ± S.D.	Mean ± S.D.	*p* value
Age (years)	27.9 ± 4.8	28.2 ± 6.1	0.785^a^
BMI (kg/m²)	23.9 ± 2.3	23.9 ± 3.6	0.997 ^a^
Parity	1.0 ± 1.1	1.0 ± 1.0	0.703 ^b^
Abortion	0.3 ± 0.6	0.5 ± 0.9	0.259 ^b^
Gestational Age (weeks)	9.9 ± 2.4	9.8 ± 2.4	0.838 ^b^
PUQE Score	2.7 ± 1.7	10.2 ± 1.9	**< 0.001** ^**b**^
WBC (×10⁹/L)	8.2 ± 1.4	12.5 ± 0.8	**< 0.001** ^**b**^
Neutrophil (×10⁹/L)	5.2 ± 1.2	8.2 ± 0.8	**< 0.001** ^**b**^
Lymphocyte (×10⁹/L)	1.9 ± 0.4	1.8 ± 0.3	0.086 ^a^
Monocyte (×10⁹/L)	0.5 ± 0.2	0.6 ± 0.1	**< 0.001** ^**a**^
Platelet (×10⁹/L)	269.7 ± 48.7	323.3 ± 37.2	**< 0.001** ^**a**^
Hemoglobin (g/dL)	12.0 ± 0.7	11.6 ± 0.8	**0.006** ^**b**^
Hematocrit (%)	34.5 ± 1.6	32.8 ± 2.4	**< 0.001** ^**b**^
Sodium (mmol/L)	139.5 ± 2.3	137.5 ± 3.1	**< 0.001** ^**b**^
Potassium (mmol/L)	4.2 ± 0.3	4.0 ± 0.4	**0.028** ^**a**^
Chloride (mmol/L)	103.1 ± 1.9	102.4 ± 2.7	0.156 ^b^
AST (U/L)	19.5 ± 4.6	23.8 ± 6.2	**< 0.001** ^**a**^
ALT (U/L)	19.0 ± 6.4	23.0 ± 7.9	**0.013** ^**a**^
LDH (U/L)	203.6 ± 25.5	236.1 ± 48.9	**< 0.001** ^**a**^
NLR	2.9 ± 0.99	4.83 ± 1.28	**< 0.001** ^**a**^
MLR	0.27 ± 0.11	0.35 ± 0.08	**< 0.001** ^**a**^
PLR	149.2 ± 49.9	189.9 ± 51.5	**< 0.001** ^**a**^
SII	784.3 ± 337.5	1585.5 ± 544.7	**< 0.001** ^**b**^

a: Independent samples *t-test*, b: Mann–Whitney *U* test. Data are presented as mean ± standard deviation (SD). *PUQE* Pregnancy-Unique Quantification of Emesis, *WBC* white blood cell, *NLR* neutrophil-to-lymphocyte ratio, *MLR* monocyte-to-lymphocyte ratio, *PLR* platelet-to-lymphocyte ratio, *SII* systemic immune-inflammation index

Spearman correlation analysis demonstrated significant positive associations between PUQE score and NLR (ρ = 0.677, *p* < 0.001), MLR (ρ = 0.588, *p* < 0.001), PLR (ρ = 0.601, *p* < 0.001), and SII (ρ = 0.705, *p* < 0.001). Similarly, urinary ketone levels were strongly correlated with NLR (ρ = 0.853), MLR (ρ = 0.691), PLR (ρ = 0.827), and SII (ρ = 0.899) (all *p* < 0.001). These findings indicate that increasing inflammatory burden parallels both clinical severity and metabolic deterioration in hyperemesis gravidarum (Table 2).

**Table 2:** Correlation of PUQE and Ketonuria with Systemic Inflammatory Indices in HG

		PUQE Score	Urine Ketone
NLR	Spearman’s rho	0.677	0.853
	Sig. (2-tailed)	**< 0.001**	**< 0.001**
	N	40	40
MLR	Spearman’s rho	0.588	0.691
	Sig. (2-tailed)	**< 0.001**	**< 0.001**
	N	40	40
PLR	Spearman’s rho	0.601	0.827
	Sig. (2-tailed)	**< 0.001**	**< 0.001**
	N	40	40
SII	Spearman’s rho	0.705	0.899
	Sig. (2-tailed)	**< 0.001**	**< 0.001**
	N	40	40

Correlation analysis was performed using Spearman correlation test. *PUQE *Pregnancy-Unique Quantification of Emesis, *NLR *neutrophil-to-lymphocyte ratio, *MLR *monocyte-to-lymphocyte ratio, *PLR *platelet-to-lymphocyte ratio, *SII *systemic immune-inflammation index

No significant differences were found in age, BMI, parity, abortion history, gestational age, or electrolyte parameters between the two subgroups (*p* > 0.05). However, hospitalized patients had significantly higher PUQE scores (11.1 ± 1.4 vs. 8.1 ± 0.8, *p* < 0.001), WBC, neutrophil, monocyte, and platelet counts (all *p* < 0.001), and markedly lower lymphocyte levels (*p* < 0.001). Consequently, NLR, MLR, PLR, and SII values were all substantially higher in the hospitalized group (all *p* < 0.001), suggesting that these indices are predictive of disease severity and the need for inpatient management (Table 3).

**Table 3:** Clinical and Laboratory Predictors of Hospitalization in HG

	Hospitalisation (-) (*n* = 19)	Hospitalisation (+) (*n* = 21)	
	Mean ± S.D.	Mean ± S.D.	*p* value
Age (years)	26.9 ± 7.0	28.8 ± 5.7	0.362^a^
BMI (kg/m²)	24.9 ± 4.0	23.5 ± 3.4	0.239 ^a^
Parity	1.3 ± 1.2	0.9 ± 1.0	0.328^b^
Abortion	0.3 ± 0.7	0.6 ± 1.0	0.652 ^b^
Gestational Age (weeks)	10.7 ± 2.1	9.4 ± 2.5	0.108 ^b^
PUQE Score	8.1 ± 0.8	11.1 ± 1.4	**< 0.001** ^**b**^
WBC (×10⁹/L)	11.7 ± 0.9	12.9 ± 0.5	**< 0.001** ^**b**^
Neutrophil (×10⁹/L)	7.3 ± 0.5	8.6 ± 0.5	**< 0.001** ^**b**^
Lymphocyte (×10⁹/L)	2.0 ± 0.3	1.7 ± 0.2	**< 0.001** ^**a**^
Monocyte (×10⁹/L)	0.5 ± 0.1	0.6 ± 0.1	**< 0.001** ^**a**^
Platelet (×10⁹/L)	293.5 ± 28.7	336.1 ± 33.1	**< 0.001** ^**a**^
Hemoglobin (g/dL)	11.8 ± 1.1	11.5 ± 0.7	0.328 ^b^
Hematocrit (%)	33.3 ± 2.8	32.6 ± 2.1	0.631 ^b^
Sodium (mmol/L)	137.4 ± 3.6	137.5 ± 2.9	0.805 ^b^
Potassium (mmol/L)	4.0 ± 0.5	4.0 ± 0.3	0.741 ^a^
Chloride (mmol/L)	102.0 ± 1.4	102.5 ± 3.0	0.550 ^b^
AST (U/L)	23.2 ± 4.7	24.0 ± 6.8	0.717 ^a^
ALT (U/L)	22.2 ± 8.2	23.4 ± 7.9	0.659 ^a^
LDH (U/L)	250.6 ± 66.3	229.9 ± 39.1	0.225 ^a^
NLR	3.68 ± 0.47	5.33 ± 1.19	**< 0.001** ^**a**^
MLR	0.27 ± 0.05	0.38 ± 0.07	**< 0.001** ^**a**^
PLR	149.0 ± 27.5	207.4 ± 49.7	**< 0.001** ^**a**^
SII	1080.5 ± 192.3	1802.0 ± 501.6	**< 0.001** ^**b**^

a: Independent samples *t*-test, b: Mann–Whitney *U* test. Data are presented as mean ± standard deviation (SD).* PUQE *Pregnancy-Unique Quantification of Emesis, *WBC *white blood cell; NLR: neutrophil-to-lymphocyte ratio*, MLR *monocyte-to-lymphocyte ratio*, PLR *platelet-to-lymphocyte ratio*, SII *systemic immune-inflammation index

**Fig. 1:**
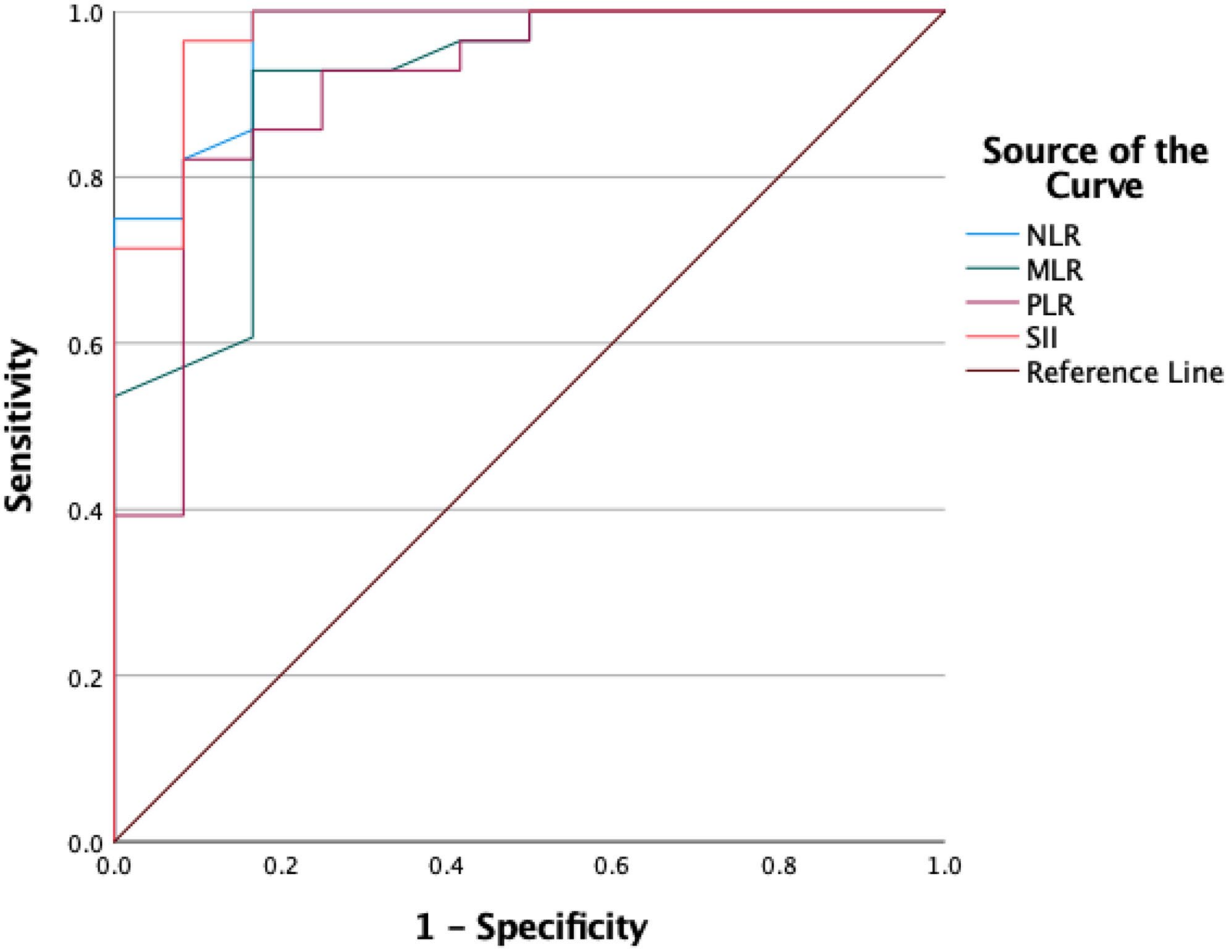
ROC Curves of Systemic Inflammatory Indices (NLR, MLR, PLR, And SII) for Predicting Hospitalization in Patients with HG

Of the 40 patients with hyperemesis gravidarum, 21 (52.5%) required hospitalization, whereas 19 (47.5%) were managed on an outpatient basis. Hospitalization decisions were made according to predefined clinical criteria, including dehydration, electrolyte imbalance, significant ketonuria, and inability to tolerate oral intake, and were not based solely on PUQE score. SII had the highest diagnostic performance with an AUC of 0.973 (95% CI: 0.924–1.000), followed by NLR (0.966), MLR (0.909), and PLR (0.908). The optimal cut-off values for predicting hospitalization were ≥ 4.34 for NLR, ≥ 0.325 for MLR, ≥ 170.9 for PLR, and ≥ 1402.6 for SII (all *p* < 0.001). These results demonstrate that systemic inflammatory indices, particularly SII and NLR, are strong predictors of hospitalization in patients with hyperemesis gravidarum (Table 4; Fig. 1).

**Table 4:** ROC Analysis of Systemic Inflammatory Indices in Predicting Hospitalization Among Patients with HG

	AUC	Sensitivity	Specificity	Cut-off	*p* value	Asymp 95% CI	
						Lower Bound	Upper Bound
**NLR**	0.966	89	84	4.34	**< 0.001**	0.913	1.000
**MLR**	0.909	79	87	0.325	**< 0.001**	0.808	1.000
**PLR**	0.908	82	88	170.9	**< 0.001**	0.801	1.000
**SII**	0.973	86	91	1402.6	**< 0.001**	0.924	1.000

*AUC *area under the curve, *CI *confidence interval, *NLR *neutrophil-to-lymphocyte ratio, *MLR *monocyte-to-lymphocyte ratio, *PLR *platelet-to-lymphocyte ratio, *SII *systemic immune-inflammation index. ROC analysis was used to determine optimal cut-off values of inflammatory indices for predicting hospitalization in patients with hyperemesis gravidarum

In multivariable linear regression analysis adjusted for maternal age, body mass index, and gestational age, both SII and NLR remained significantly associated with PUQE score within the HG group. Specifically, each 100-unit increase in SII was associated with a 0.231-point increase in PUQE score (95% CI: 0.149–0.313; *p* < 0.001), and each one-unit increase in NLR corresponded to a 0.877-point increase in PUQE score (95% CI: 0.492–1.262; *p* < 0.001) (Table 5).

**Table 5:** Multivariable Linear Regression Analysis of Factors Associated with PUQE Score in Patients with Hyperemesis Gravidarum

	β (Unstandardized Coefficient)	95% Confidence Interval	*p*-value
SII (per 100 units)	0.231	0.149–0.313	**< 0.001**
NLR (per 1 unit)	0.877	0.492–1.262	**< 0.001**

β: unstandardized regression coefficient. Model adjusted for maternal age, body mass index (BMI), and gestational age. *PUQE *Pregnancy-Unique Quantification of Emesis, *SII *systemic immune-inflammation index, *NLR *neutrophil-to-lymphocyte ratio.

## Discussion

The present study revealed that systemic inflammatory indices — including the neutrophil-to-lymphocyte ratio (NLR), monocyte-to-lymphocyte ratio (MLR), platelet-to-lymphocyte ratio (PLR), and systemic immune-inflammation index (SII) — were significantly higher in patients with hyperemesis gravidarum (HG) than in healthy controls. Furthermore, these indices showed strong positive correlations with PUQE scores and urinary ketone levels, indicating that the degree of inflammatory activation parallels the clinical and metabolic severity of HG. Among these indices, SII and NLR demonstrated the highest diagnostic accuracy in predicting hospitalization, suggesting their potential as simple, cost-effective markers for assessing disease severity. Unlike prior studies primarily comparing HG and control groups, our analysis specifically quantified the relationship between inflammatory burden and standardized PUQE-based severity grading, thereby providing an objective severity-linked inflammatory profile.

Hyperemesis gravidarum is a multifactorial disorder in which inflammatory and immunological mechanisms are increasingly recognized as central components of its pathophysiology. Physiological pregnancy is characterized by a carefully regulated inflammatory milieu that supports implantation and placental development; however, an excessive or dysregulated inflammatory response may contribute to pathological conditions such as HG [12]. Negishi and Morita emphasized that disruption of the balance between proinflammatory and anti-inflammatory mediators during early gestation can trigger systemic immune activation and oxidative stress [7]. In parallel, Singh et al. reported in a large meta-analysis that altered maternal immune regulation and cytokine imbalance are implicated in several pregnancy-related complications, further supporting the immunological basis of HG [5].

Consistent with previous literature, our study observed that patients with HG exhibited higher neutrophil and platelet counts and lower lymphocyte levels, reflecting an enhanced proinflammatory state. This is in agreement with Uçkan et al. and Tayfur et al., who reported significant elevations in NLR and PLR among HG patients compared with healthy pregnant women [6, 13]. The same trend was observed by Bayram [14] and Beşer [15], who found that SII — a composite index incorporating neutrophil, platelet, and lymphocyte counts — was significantly increased in HG and correlated with disease severity. The elevation of SII in our cohort may reflect the synergistic effect of neutrophilia and thrombocytosis accompanying lymphopenia, a profile commonly observed in systemic inflammation.

Moreover, we observed a strong correlation between PUQE score and inflammatory indices, suggesting that the intensity of nausea and vomiting parallels the degree of systemic inflammatory activation. Importantly, in multivariable linear regression analysis adjusted for maternal age, body mass index, and gestational age, both SII and NLR remained significantly associated with PUQE score. This finding indicates that the relationship between inflammatory burden and clinical severity is not fully explained by basic maternal characteristics. However, given the observational design and the relatively modest sample size, these associations should be interpreted cautiously and should not be construed as evidence of causality. Previous studies have reported significant associations between inflammatory markers and both ketonuria and disease severity in HG, including elevations in NLR, PLR, and other neutrophil-based indices [16–18]. These findings collectively reinforce the link between metabolic stress, immune activation, and increasing clinical severity in hyperemesis gravidarum.

In contrast, Emekçi and Özay [19] reported no significant differences in NLR and PLR between HG and controls, underscoring the heterogeneity of findings across studies. Such variability may reflect differences in study design, sampling timing, and patient selection. Despite these inconsistencies, the cumulative evidence favors an inflammatory component in HG. In our cohort, SII and NLR demonstrated excellent discriminatory performance for predicting hospitalization (AUC = 0.973 and 0.966, respectively), consistent with previous reports [14, 15]. Their clinical utility is further supported by their simplicity, reproducibility, and availability within routine laboratory testing. Although the observed AUC values were high, these estimates were derived from a relatively small single-center cohort and may be subject to optimism bias. The cut-off values were internally generated and have not undergone external validation. Therefore, the reported discriminatory performance should be interpreted cautiously, and independent validation in larger, multicenter populations is required before clinical implementation.

The pathophysiological mechanisms linking systemic inflammation and HG remain incompletely understood; however, elevated proinflammatory cytokines such as TNF-α, IL-6, and IL-8 have been associated with gastrointestinal symptoms and central nausea pathways [5, 7]. Cytokine-mediated neuroinflammatory signaling, together with hormonal interactions involving human chorionic gonadotropin (hCG) and estrogen, may exacerbate nausea and vomiting severity. Jansen et al. [3] further suggested that inflammatory activation may contribute not only to symptom persistence but also to dehydration and metabolic deterioration requiring hospitalization. Within this biological framework, our findings underscore the potential clinical value of integrating hematological inflammatory indices into routine assessment of HG. In particular, SII—previously shown to differentiate inflammatory states in various endocrine and systemic disorders [9] —may serve as a practical, accessible biomarker to identify patients at increased risk for severe disease or hospitalization.

The relationship between inflammatory burden and hospitalization in hyperemesis gravidarum has been only partially explored. Balkaş et al. demonstrated that albumin-based immuno-nutritional indices, particularly m-HALP, were independently associated with re-hospitalization risk and length of stay, underscoring the prognostic relevance of composite markers in HG. In contrast, our study evaluated hematologic inflammation-dominant indices derived from routine complete blood counts [20] and demonstrated that SII and NLR showed high discriminatory capacity for predicting index hospitalization. Although the biological substrates of these indices differ, both lines of evidence converge on the concept that heightened systemic inflammatory activation is linked to clinically significant deterioration in HG. Nevertheless, differences in study design and outcome definitions (index hospitalization versus re-hospitalization) warrant cautious interpretation. Moreover, hospitalization as an outcome may vary according to institutional admission thresholds and clinical practice patterns, potentially limiting external validity and generalizability of these findings.

Despite these promising results, several limitations should be acknowledged. This was a single-center study with a relatively small cohort, which may limit the generalizability of findings. Inflammatory indices were measured at a single time point, without follow-up to assess their dynamic change with clinical improvement. Moreover, classical inflammatory biomarkers such as C-reactive protein (CRP) and interleukin-6 (IL-6) were not evaluated, precluding direct comparison between hematological indices and established inflammatory markers. Hospitalization, although predefined by objective clinical criteria, may still be influenced by clinician judgment and institutional practice patterns, potentially introducing decision-related variability. In addition, factors such as dehydration-related hemoconcentration, nutritional status, and acute physiological stress—which may affect hematological inflammatory indices—were not independently quantified or adjusted for in the analyses. Although multivariable models were applied for HG status and PUQE-based severity, residual confounding cannot be entirely excluded. Furthermore, given the observational case–control design, the findings should be interpreted strictly as associations rather than evidence of causality. Future multicenter studies with standardized admission criteria, longitudinal assessment, and broader clinical adjustment are warranted to validate these observations.

In conclusion, our findings support the growing body of evidence indicating that systemic inflammatory activation is associated with the clinical expression of hyperemesis gravidarum. Among the evaluated hematological indices, SII and NLR demonstrated the strongest associations with standardized PUQE-based disease severity and showed high discriminatory performance for index hospitalization within this cohort. Rather than proposing a novel inflammatory mechanism, this study provides a structured integration of routinely available hematological indices with standardized severity assessment in a prospective analytical framework. The observed performance of SII suggests potential incremental clinical utility in identifying patients at increased risk for more severe disease and inpatient management. Given their accessibility, low cost, and reproducibility, these indices may serve as supportive markers within clinical risk stratification strategies. However, the findings should be interpreted as associative, and external validation in larger, multicenter cohorts with longitudinal follow-up is necessary before broader clinical implementation.

## Data Availability

The datasets generated and analyzed during the current study are available from the corresponding author upon reasonable request.
